# Determinants of adherence to COVID-19 measures among the Belgian population: an application of the protection motivation theory

**DOI:** 10.1186/s13690-021-00565-9

**Published:** 2021-05-12

**Authors:** Joris Adriaan Frank van Loenhout, Kirsten Vanderplanken, Bénédicte Scheen, Stephan Van den Broucke, Isabelle Aujoulat

**Affiliations:** 1 Centre for Research on the Epidemiology of Disasters (CRED), Institute of Health and Society, Université catholique de Louvain, Clos Chapelle-Aux-Champs 30, 1200, Brussels, Belgium; 2 Centre for Health promotion knowledge transfer (RESO), Institute of Health and Society, Université catholique de Louvain, Brussels, Belgium; 3 Psychological Sciences Research Institute, Université catholique de Louvain, Louvain-la-Neuve, Belgium

**Keywords:** COVID-19, Protection motivation Theory, Adherence, Perceived severity, Vulnerability, Response efficacy, Self-efficacy

## Abstract

**Background:**

Since the start of the COVID-19 outbreak, the Belgian government has implemented various infection prevention and control measures. This study assessed the extent to which the general population in Belgium adhered to these measures, and which determinants were associated with adherence.

**Methods:**

We undertook an internet survey among a sample of the Belgian population, representative for sex, age, socio-economic status and province. The questionnaire included various demographic, socio-economic and health-related questions, and also drew upon the Protection Motivation Theory as a theoretical framework to measure levels of perceived severity, vulnerability, perceived usefulness of the measures (response efficacy), perceived personal capacity to adhere (self-efficacy), and past and future adherence. Data were collected in Dutch and French, the main languages of Belgium.

**Results:**

Our study was carried out in September 2020, and the number of respondents was 2008. On average, respondents provided high scores for each of the measures in place in September in terms of response efficacy (range of 3.54–4.32 on 1 to 5 Likert-scale), self-efficacy (range of 3.00–4.00), past adherence (4.00–4.68) and future adherence (3.99–4.61). The measure that overall received the highest scores was wearing a face mask in public spaces, while ‘the social bubble of 5’ generally received the lowest scores. There was a statistically significant relationship between response efficacy and self-efficacy on the one hand and (past and future) adherence on the other hand, in a multivariate model corrected for confounders. Vulnerability and severity did not show statistical significance.

**Conclusion:**

Risk communication regarding COVID-19 should place a stronger emphasis on helping people understand why implemented measures are useful and how they can be put into practice, more than on increasing fear appeals.

## Background

The outbreak in 2020 of the novel coronavirus SARS-CoV-2, which leads to the contagious disease COVID-19, has become a pandemic without precedent. After it was first detected in December 2019 in Wuhan, China, the virus spread quickly over the world. In Belgium the first case was detected on February 3^rd^ 2020. By the 12^th^ of January 2021, 665,223 cases, 49,359 hospital admissions and 20,122 deaths had been reported [1]. Thus far, two peaks in the outbreak have occurred (in March–April and October–November 2020), and recurrent peaks in infections are likely to occur later in time. Until a sufficient proportion of the public has been vaccinated or is immune, it must be ensured that the gross majority of the public adheres to infection prevention and control measures (IPCM) that limit the spread of COVID-19 [2].

The Belgian government has implemented various IPCM from February 1^st^ 2020 onwards [3]. Mathematical modelling of transmission dynamics shows that the success and impact of these IPCM relies heavily on the public’s adherence to them [4, 5]. Perceptions of the threat posed by COVID-19 to individuals, their families and friends, and to society at large, are key to motivating behaviour change [6, 7]. To enhance the efficiency and effectiveness of adherence to IPCM, a better understanding is needed about what citizens know about the IPCM, whether they understand the measures that are imposed by the authorities, how they perceive these measures and COVID-19-related risks, and whether they adhere to the measures [8]. In this respect, there is a shortage of population-based studies at a national level in Belgium that capture such insights.

Many of the measures issued by the Belgian government include communication about the risks posed by the virus, since it is a general assumption that better knowledge and understanding of these risks will lead to greater adherence [9]. A poor understanding regarding COVID-19 infection and transmission can indeed hinder the adoption of protective behaviours and risk recognition [10]. Furthermore, individuals who perceive the recommended or prescribed measures to be clear and consistent show greater adherence than those who perceive them as less clear or consistent [11]. A lower level of understanding about the disease may also result in individuals consulting fewer information sources and distrusting COVID-19 information [10]. In Belgium, it has been suggested that a low overall level of trust in governmental institutions and a lack of consensus among politicians and scientists during this crisis, have led to lower motivation of the public to comply strictly with the preventive measures [12, 13]. Unfortunately, the abundance of information that is available about COVID-19 through different channels, some of which is misleading, biased or incorrect, does not make it easy for the public to identify scientific evidence and reliable sources. This may have adverse consequences for public health [14]. A possible strategy to deal with these challenges is to identify credible sources for delivering evidence to different audiences, and to then adjust the messaging strategy according to the target group. For instance, the emphasis can be put on the benefits for the recipient or on protecting others, and an appeal can be made to moral values or social norms, based on the audience’s motivation [14, 15].

However, adherence to the proposed measures cannot be achieved by informing people about the risks posed by the virus alone. Like most human behaviour, adhering to IPCM is not a rational behaviour. Even if people feel that adhering to IPCM is effective in limiting the success of the COVID-19 spread, this does not guarantee overall adherence. Hence, other determinants of protective behaviour should also be addressed. These determinants are specified in health psychological models, such as the Health Belief Model [16] or the Protection Motivation Theory [17–19]. According to the latter, people will only act on health warnings and adopt precautionary measures if they (i) perceive a threat to be severe, (ii) consider themselves personally susceptible or vulnerable to develop the condition, (iii) believe that the recommended protective behaviour is effective, and (iv) consider themselves capable to perform the behaviour.

Research on how the public reacts to IPCM that are implemented or recommended by governments across the world confirms that adherence to IPCM is related to a high level of *perceived risk* of COVID-19. Individuals who perceive greater impact of an infection with the virus are more likely to adopt IPCM as an avoidance strategy [20–22]. In turn, the level of perceived risk depends on a variety of factors, including personal experience with the virus, having prosocial values, and trust in science and medical practitioners [22]. In a similar vein, the level of *perceived vulnerability* to COVID-19 has been found to influence how people consider the necessity of abiding by IPCM and to increase adherence [23–25]. Other studies confirm the role of *perceived behavioural control* or self-efficacy in complying with IPCM in the context of COVID-19. In general, people who feel capable and who have the resources to perform the protective measures show better adherence to them [21, 23, 26], although it also seems that perceived control influences adherence to different measures in varying degrees [27]. Lastly, the perceived efficacy of the recommended or mandatory measures (also known as *response efficacy*) has also been investigated. Understanding the necessity and value of a particular measure, and considering it as legitimate, appears to increase the voluntary adherence to those measures [28]. Likewise, a positive attitude regarding the measures and the belief that they actually help in the fight against the virus, are linked with higher adherence to IPCM [27]. In this relation between perceived efficacy and adherence, self-efficacy has shown to be an important mediating factor [26].

In Belgium, several studies have investigated the factors that contribute to IPCM adherence, using diverse sampling methods and analysis models and covering different region of the country [27, 29]. However, a limitation is that they are not representative for the entire Belgian population. The only studies so far that provided data on a range of COVID-19 related issues at national level are the surveys performed by the national Public Health Institute Sciensano [1], yet these surveys were not informed by behavioural theories on protective behaviour. This study fills that gap by drawing on the Protection Motivation Theory (PMT) as a theoretical framework to measure levels of threat appraisal and coping appraisal towards COVID-19 and IPCM among a large, representative sample of the Belgian population, and assessing the relationship with individual past and future adherence to IPCM.

## Methods

### Questionnaire

Data were collected online using a survey comprised of five sections. Section 1 contained a series of questions on demographic and socio-economic characteristics (gender, age, province, professional situation, education, household composition and household income). Section 2 contained questions about the respondent’s health status (perceived health, dependency on care or help from others, having others being dependent on the respondent’s help or care). Section 3 consisted of questions about the participant’s experience with COVID-19 (tested positive) and perceived consequences of contracting the disease (perceived severity and vulnerability). To measure perceived severity, respondents who had not been ill and had not tested positive for COVID-19 were asked to rate the expected health consequences on a scale of 0–100 (0 = ‘not at all severe’, 100 = ‘very severe’). For respondents who had shown symptoms and/or had tested positive, the question was modified to assess the experienced, rather than expected, severity of the disease. Perceived vulnerability was measured by having respondents score the risk for themselves and significant others (parents, grandparents, partners, children, friends, colleagues) on 5-point Likert scales (0 = ‘no risk at al’, 5 = ‘definite’). Section 4 (the results of which are not reported in this paper) consisted of 5 questions asking respondents about their use of information sources to obtain information about COVID-19 and about the protective measures, and on the perceived understandability and trustworthiness of these sources. These were also scored on 5-point Likert scales (0 = ‘not at all’, 5 = ‘very much’). Section 5 was composed of questions related to protective behaviour, i.e., the self-reported understanding of eight key IPCM that were launched in August and still applicable in September (Appendix 1), the perceived usefulness (response efficacy) of the measures, the perceived difficulty to adhere to the measures (self-efficacy), and past and intended adherence to the measures. All were to be scored on 5-point Likert scales (0 = ‘not at all useful/easy/adhered to’, 5 = ‘very useful/easy/adhered to’).

The survey was first developed in English, which was the common language within the research team, and then translated to Dutch and French, and back-translated in order to check the accuracy of the translation. The questionnaire is available as additional material (Appendix 2).

### Study participants

To participate in this study, a sample of around 2000 participants was drawn from an online panel. Participants were only accepted to the sample if they fitted within the pre-defined demographic and socio-economic quotas, fully completed the survey, and passed certain quality controls. By ensuring that all participants fitted within the pre-defined quotas, the representativeness of the sample for the Belgian population between the ages of 18–75 years was guaranteed. The demographic quotas were based on gender, age and province/region of residence. Regarding socio-economic criteria, 8 subgroups were defined, to be represented equally in the survey. This grouping was based on the respondents’ level of education, profession, and current working status. All panel members were proficient in Dutch and/or French.

### Data collection

Data collection was organised via a specialised market research and opinion poll company. A first group of potential respondents was selected from the panel, based on the aforementioned criteria, and invited via e-mail to participate in the survey. Those who proceeded to participate in the survey received a personalized login that allowed them to quickly access the online survey, ensuring that each respondent could only complete the survey once. After this first round, the sample was gradually completed by (re-)contacting panel members who fit those quotas that were not yet completed. The final sample was representative of the Belgian population in terms of the pre-defined criteria.

Quality controls were applied at three points in time to ensure the quality of responses provided by respondents. Before the survey was launched, the programming and encoding of the data were verified. During the survey, quality control questions were inserted within the survey (e.g. “To ensure that you complete the questionnaire correctly, please enter the number 7”) to identify and dismiss inattentive respondents. Finally, during the period in which the survey data were collected, the system automatically monitored completion time (respondents who completed the survey at least 50% below the average time were eliminated) and answer patterns (respondents who systematically gave the same scores were eliminated). The number of completed surveys was monitored on a daily basis to ensure there were no technical or other issues. Finally, at the end of the data collection phase, the quality and consistency of the answers were controlled.

The survey was pilot tested by the survey company on 7 September 2020 on a pre-sample of 50 respondents. Their answers were provided in the same format as the final dataset. An additional pilot test was performed by the research team between 4 and 7 September 2020, whereby several people were asked to complete the survey and provide feedback on any issues they encountered. Based on both pilot tests, minimal changes were made to improve the survey.

The data were collected between 7 and 24 September 2020. New protective measures against COVID-19 were announced on September 23^rd^. As these could have impacted the response to a small number of the questions in the survey, the responses to these specific questions were removed from files completed as of that date, which concerned around 6% of respondents. Responses to questions that were not affected by the new measures were retained.

### Data analysis

All analyses were performed using IBM SPSS Statistics 25. Descriptive overviews were presented of all variables. For continuous variables and questions with Likert scale, average scores were calculated, as well as the standard deviations. Questions from sections 3 and 5 were used to operationalize the dimensions of the Protection Motivation Theory (perceived severity of the threat, perceived vulnerability, response efficacy, self-efficacy) and for past and future adherence to the IPCM. Apart from perceived severity, which consisted of a single score, this was done by averaging the scores on the Likert scales for the items belonging to each dimension. Internal consistencies for the scales that thus obtained were verified by Cronbach’s alphas, which were all higher than .800 (Appendix 3).

To investigate the degree to which the components of the PMT were associated with past and future adherence to the measures, a multivariate regression analysis was performed with the PMT components as independent variables (uncorrected model). In a second analysis, a number of demographic, socio-economic and health characteristics were added as confounders (corrected model).

## Results

### Participation and respondent characteristics

Invitations for the survey were sent to approximately 22,000 potential respondents. In total 3257 respondents started the survey. Out of those, 941 respondents were not accepted because quota for their socio-demographic group were full; 177 dropped out (started the survey but did not finish); 131 were refused because their survey was of insufficient quality. This leaves the final number of respondents at 2008. Of these, 1135 were Dutch-speaking (56.5%) and 873 French-speaking (43.5%). The proportion of females was slightly higher than the proportion of men (51% vs. 49%) (Table 1), reflecting the composition of the Belgian population and thus matching the predefined demographic quota. All age groups were represented more or less equally, until 75 years of age. The proportion of respondents per region was also similar to the distribution of the Belgian population. In terms of education level, the largest group of respondents (over 40%) were those with higher secondary education. In terms of occupation, almost half of respondents had an occupation, and more than a quarter was pensioned. In terms of income, most respondents fell within the two average categories.

**Table 1:** Demographic and socio-economic characteristics of study population, and experience with COVID-19

Characteristic	N	%
Gender		
Male	983	49.0
Female	1024	51.0
Other	1	0.0
Age		
18–30 years	407	20.3
31–45 years	472	23.5
46–60 years	522	26.0
61–75 years	557	27.7
76 years and over	50	2.5
Region		
Flanders	1140	56.8
Wallonia	662	33.0
Brussels	206	10.3
Educational level		
Primary or without diploma	62	3.1
Lower secondary	240	12.0
Upper secondary	810	40.3
Superior short type and bachelors	420	20.9
Long/university level superior	471	23.5
Occupation		
Yes	920	45.8
No, incapacitated to work	161	8.0
No, pre-pension	33	1.6
No, pension	530	26.4
No, unemployed	80	4.0
No, student	180	9.0
No, homemaker	88	4.4
No, never or not yet worked	16	0.8
Net annual household income		
Less than EUR 15,000	164	8.2
Between EUR 15,000 and 29,999	612	30.5
Between EUR 30,000 and 44,999	534	26.6
More than 45,000	319	15.9
I do not know	379	18.9
Tested positive for COVID-19		
Not tested positive and no COVID-19 symptoms	1709	85.1
Not tested positive but had COVID-19 symptoms	198	9.9
Tested positive but without COVID-19 symptoms	41	2.1
Tested positive for COVID-19 symptoms and hospitalised	1	0.0
Tested positive for COVID-19 symptoms but no hospitalisation	26	1.3
Don’t know if tested positive for COVID-19	33	1.6

### Experience with COVID-19

At the moment of the survey, a large majority of respondents (*n* = 1742) had not been diagnosed as infected with COVID-19, and had not shown any symptoms possibly indicating an infection (Table 1). Approximately one out of ten participants (*n* = 199) had experienced symptoms that could indicate COVID-19, but had not been tested (positive), while 42 respondents had tested positive but had shown no symptoms. Only 27 participants had a confirmed COVID-19 infection with symptoms, one of whom had been hospitalised. Those who had (possibly) been infected with COVID-19 were generally younger (41.7 vs. 49.4 years) and higher educated (Chi square, *p* = .002) than those who had not had COVID-19, nor symptoms of infection.

### Perceived risk

Respondents who had not been ill and had not tested positive for COVID-19 (*n* = 1709) had an average score of 57.3/100 (sd 27.4) for expected health consequences of the infection. This is higher than the average scores of 33.8/100 (sd 28.7) for the 198 respondents who had unconfirmed symptoms of COVID-19, and 33.3/100 (sd 32.3) for the 41 respondents with a positive test but no symptoms. For the 27 respondents who had had a confirmed COVID-19 infection with symptoms, the average score in terms of severity was 51.4/100 (sd 26.0).

With regard to perceived vulnerability, there is not much difference in the perceived risk of becoming infected with COVID-19 oneself compared to significant others (parents, grandparents, partners and children). The highest scores were seen for friends and colleagues (Table 2).

**Table 2:** Perceived risk of infection with COVID-19

Person(s)	N	Mean	sd
Yourself	1986	2.85	0.93
Your parents	1347	2.84	0.97
Your grandparents	735	2.60	1.09
Your partner	1467	2.90	0.94
Your child (ren)	1345	2.94	0.94
A friend	1805	3.14	0.84
A close colleague	1286	3.16	0.90

### Perceived efficacy of protective measures

The average scores for perceived usefulness, ease of adherence, and past and future adherence to each of the eight COVID-19 measures are presented in Table 3. Shopping with a maximum of one other person and restricting social contacts to a “social bubble” of five persons were considered as the least useful, as opposed to working from home and wearing a face mask, which were perceived as the most useful. On average, respondents gave lower scores to whether they considered the measures easy to adhere to than for their usefulness. The highest score for ease of adherence was observed for the measure shopping with maximum one other person, and the lowest for the measure regarding the “social bubble”, which received an average score of 3.00 on a scale of 1 to 5 (equivalent to the answer option ‘neutral’ in the questionnaire).

**Table 3:** Average scores in understanding, usefulness, ease to adhere, past adherence and future adherence for each of the 8 current COVID-19 measures

	Useful	Easy to adhere	Past adherence	Future adherence	Comparison past / future adherence
**Measure**	**Mean (sd)**	**Mean (sd)**	**Mean (sd)**	**Mean (sd)**	***p*-value**
Social bubble limited to 5	3.54 (1.39)	3.00 (1.44)	4.00 (1.26)	3.99 (1.29)	.316
Private events limited to 10	3.66 (1.34)	3.37 (1.35)	4.42 (1.04)	4.27 (1.11)	< .001
Official events limited to 200 (indoors) or 400 (outdoors)	3.63 (1.41)	3.67 (1.27)	4.58 (0.90)	4.52 (0.93)	< .001
Homeworking strongly recommended	4.32 (1.03)	3.81 (1.27)	4.08 (1.30)	4.16 (1.25)	.580
Shop with max. One other person	3.53 (1.37)	4.00 (1.20)	4.55 (0.93)	4.45 (1.01)	< .001
Wearing a face mask in public spaces	4.16 (1.22)	3.94 (1.25)	4.68 (0.74)	4.61 (0.83)	< .001
Travel form	3.88 (1.32)	3.93 (1.13)	4.40 (1.05)	4.45 (1.00)	1.00
Travel zones	3.91 (1.26)	3.75 (1.19)	4.39 (1.03)	4.48 (0.99)	.418

### Past and future adherence to protective measures

Overall, a high level of self-reported adherence to the IPCM was noted for all measures (average scores of 4.00 to 4.68 on a 5-point scale), as well as for the intention to adhere to the measures in the future (means of 3.99 to 4.61). Comparison between the adherence scores for past and future behaviour by means of paired-samples t-tests showed statistically significant differences for the measures on restricting the number of people on private and on official events, respecting the number of accompanying persons when shopping, and wearing a face mask. For all four measures, future adherence scored lower than past adherence. The measures for which both past and future adherence scored relatively high were wearing a face mask in public and limiting the number of people at official events. Measures for which both past and future adherence scored relatively low were the ones related to working from home and respecting the social bubble of five persons.

### Determinants of adherence

Multivariate regression analysis with the PMT components of perceived severity, perceived vulnerability, response efficacy and self-efficacy as independent variables and (past and future) adherence to the measures revealed that both past adherence and the intention to adhere in the future were significantly predicted by the model (respective R^2^ = .364; *p* < .001 and R^2^ = .459; *p* < .001) (Table 4). This also held true when the model was corrected for demographic, socio-economic and health related confounders (respective R^2^ = .394; *p* < .001 and R^2^ = .482; *p* < .001). There was no difference in the direction or significance level between past and future adherence. Perceived vulnerability was not significantly associated with adherence. A weak, positive relationship was found between perceived severity and adherence in the uncorrected models, which disappeared in the corrected models. Both response efficacy and self-efficacy were strongly, positively associated with past adherence and intention to adhere to the protective measures in the future.

**Table 4:** PMT Components associated with adherence

PMT item	Uncorrected				Corrected^**1**^			
	Past adherence		Future adherence		Past adherence		Future adherence	
	B-value (CI)	p-value	B-value (CI)	p-value	B-value (CI)	p-value	B-value (CI)	*p*-value
		< .001		< .001		< .001		< .001
Vulnerability	0.0 (0.0;0.0)	.612	0.0 (0.0;0.0)	.505	0.0 (0.0;0.1)	.386	0.0 (0.0;0.0)	.829
Severity	0.0 (0.0;0.1)	.013	0.0 (0.0;0.0)	.033	0.0 (0.0;0.0)	.326	0.0 (0.0;0.0)	.232
Response efficacy	0.2 (0.2;0.2)	< .001	0.3 (0.3;0.4)	< .001	0.2 (0.2;0.2)	< .001	0.3 (0.3;0.4)	< .001
Self-efficacy	0.3 (0.3;0.3)	< .001	0.3 (0.3;0.4)	< .001	0.3 (0.3;0.3)	< .001	0.3 (0.3;0.4)	< .001

*Past adherence, uncorrected model: R*^*2*^ *= .364; adjusted R*^*2*^ *= .363*

*Future adherence, uncorrected model: R*^*2*^ *= .459; adjusted R*^*2*^ *= .458*

*Past adherence, corrected model: R*^*2*^ *= .394; adjusted R*^*2*^ *= .385*

*Future adherence, corrected model: R*^*2*^ *= .482; adjusted R*^*2*^ *= .474*

*1) Confounders included in the corrected model were: sex, age, region, household composition, socio-economic group, dependent on care, score for health today, taking care of someone, previous COVID-19 infection*

## Discussion

The efficiency and effectiveness of infection prevention and control measures (IPCM) can be improved by obtaining detailed insights in the perception held by the population with regard to these measures. Due to differences between countries in culture and in the specific measures that are implemented, it is important to evaluate the situation on a country-level, and not only rely on international results. In that regard, there is a shortage of studies relating to the specific situation in Belgium, especially at a national (as opposed to regional) level, despite the fact that many of the measures are taken by the federal government. Furthermore, although most of the measures that are taken to prevent citizens and health workers from getting infected involve some form of behaviour change, these measures are seldom informed by behavioural theories on protective behaviour, and often rely on the assumption that better knowledge and understanding of the risks of COVID-19 will lead to greater adherence. Drawing on behaviour change principles and theories can increase the understanding of the factors that drive protective behaviour, and thus increase the effectiveness of measures. The present study addresses the aforementioned shortcomings by surveying a large, representative sample of Belgian citizens, to investigate their experience with COVID-19, their perceptions with regard to the severity of the disease, their personal vulnerability, their efficacy perceptions, and their adherence to the COVID-19 measures that were at that moment in place in Belgium, drawing on a well-validated health behaviour theory (the Protection Motivation Theory). This offers important insights for government stakeholders who are responsible for the implementation of measures to protect against COVID-19 in Belgium.

The findings of this study show that, overall, adherence to the COVID-19 measures was high among the Belgian population, despite the fact that the severity of the illness and the risk of getting infected or that someone close could get infected were generally not perceived as particularly high. On a scale of 1 to 5, all measures that were implemented at the time of the survey received an adherence score of at least 4. The intention to maintain adherence in the future was somewhat lower for some measures, but generally also remained high (score of 3.99 or higher). The lowest scores for past and future adherence were for the measure on the ‘social bubble of 5’, which is arguably the measure that has the largest impact on people’s everyday life.

The fact that perceived severity of and vulnerability for the illness are not the main drivers for adherence to preventive measures also shows from the results of the multivariate analysis. Of the four components of the PMT, only response efficacy (i.e., perceived usefulness of the measure) and self-efficacy (i.e., perceived personal capacity to adhere) showed a positive and significant relationship with past and future adherence to the measures, both for corrected and uncorrected analyses. This result is in line with an Australian study, which showed a positive relationship between perceived rating of effectiveness of behaviours and perceived ability to adopts social distancing strategies on the one hand, and adopting avoidance behaviours on the other hand [30]. Perceived severity and vulnerability did not show a significant relationship, in the model corrected for demographic, socio-economic and health-related confounders, which was surprising and in contrast to the results from other studies [20–22]. It is noted that risk communication often relies on fear induction [9], e.g. by emphasizing the risk of getting a disease and the dire consequences of the illness. As shown by our results, this has little impact on whether people will adhere to the measures, and this approach has been disputed more often during the COVID-19 pandemic [31]. In order to increase adherence, risk communication should instead focus more on helping people understand *why* implemented measures are useful, and *how* people can put them into practice.

There was an interesting difference between the perceived severity scores of respondents who had been previously infected with COVID-19 and those who had not, in the sense that the latter rated the (expected) severity of a (possible) infection as higher than the (experienced) severity of those who had had the disease. This suggests that people expect the experience of an infection with COVID-19 to be worse than it actually is. However, it is important to mention that the group of infected participants is not representative for all individuals with an infection, as those who had been seriously ill recently were less likely to participate in this survey, not to mention the people who died due to a COVID-19 infection.

Although the sample for this study closely matched the predefined stratification targets, indicating that it represents the Belgian population well in terms of gender, age (adult population until 75), region and socio-economic status, a group that was less well represented are those who belong to the lowest socio-economic group. This may partly be due to the method used: obtaining a sufficient number of responses from this particular group is often problematic in surveys, as socio-economically deprived people do not always have easy access to internet, may have more difficulty understanding a survey, and sometimes suffer from a feeling of inferiority (‘my opinion is not important’). Nonetheless, despite the lower number, this group was represented in our sample. Another limitation of performing an internet survey is that certain groups of the population are by definition underrepresented. This includes for instance school-aged young people, elderly above age 75, persons with a migrant background, and people from the informal sector (e.g. asylum seekers, sex workers). These groups typically score low on health literacy, or the cognitive and social skills to gain access to, understand, evaluate and use information in ways which promote and maintain good health [32]. Since, as in other European countries, up to 40% of the Belgian population has limited or inadequate health literacy, authorities should adapt the information about COVID-19 and the measures to protect against the virus to the literacy needs of citizens, and pay particular attention to those who are the most vulnerable in pandemics, such as elderly, migrants or people with disabilities [2]. Moreover, since our survey was only available in Dutch and French, residents in Belgium who speak another language were also excluded. This was the case, for example, for the expat population, which given the international character of the Brussels Capital Region is an important population. On the other hand, expats and other groups may use other information channels to stay informed about COVID-19 measures than the average Belgian citizen. In that respect, a specific study of these groups would be worth investigating.

Another point to mention is that the survey was undertaken in September 2020, during a time when the measures were lighter compared to other periods in the year, and when relatively few people had yet had a personal experience with COVID-19. Since October 2020, Belgium has entered a second wave, resulting in a high case load and stricter measures (e.g. ‘personal contacts limited to 1 person outside the household’ instead of a ‘social bubble of 5’). It would be of interest to retake this survey at a later stage, when different measures apply and more people have become infected, to see to what extent this influences their intention to adhere to the measures.

## Conclusion

Respondents score high with respect to past and future adherence to the COVID-19 measures in Belgium. The measure related to the ‘social bubble of 5’ has the lowest adherence, although still 4 on a scale of 1 to 5. Adherence to measures is strongly influenced by perceived usefulness of the measures, and perceived personal capacity to adhere. In contrast, perceived severity and vulnerability did not show an association with adherence. This influences how risk communication regarding COVID-19 should ideally be performed, with a stronger emphasis on helping people understand why implemented measures are useful and how they can be put into practice, more than on increasing fear appeals.

## Data Availability

The dataset used during the current study are available from the corresponding author on request.

## Appendix

### Overview of prevention and control measures implemented in Belgium – August/September 2020

A. Transport and travel
  i. In Belgium:
    - You are allowed to move around freely;
    - You have to wear a face mask or a scarf to cover mouth and nose when using public transport and above 12 years of age;
  ii. On holiday abroad
    - You are only allowed to visit countries in the EU, the UK, Switzerland, Liechtenstein, Iceland or Norway, with the exception of territories designated as red zones;
    - Follow the rules applicable in the country you are in
    - There are 3 types of travel zones:
      - You are allowed to travel to green zones;
      - You are advised not to travel to orange zones;
      - You are not allowed to travel to red zones;
    - You must fill in a passenger locator form within 48 h before returning/travelling to Belgium (see Appendix 1)
B. Work
  - Work from home if possible;
  - If you have to go into work, your employer must ensure that you are able to maintain a distance of 1.5 m from others;
  - If you can’t maintain a distance of 1.5 m from others, other measures apply that can be consulted in the guideline of the FPS Employment, Labour and Social Dialogue;
C. Shops and catering industry
  - All shops are open:
    - From August 24th you can go shopping with a maximum of 2 persons;
    - Night shops are open until 10 pm;
    - Wearing a face mask is mandatory;
    - Guidelines for shop owners;
  - Pubs and restaurants are open until 1 am:
    - It is recommended to make a reservation;
    - You can visit bars and restaurants only with your family (or the people you live with) and your bubble of 5;
    - Stay seated at your table;
    - Wearing a face mask is mandatory;
    - You must leave your contact details;
    - Guideline for owners of pubs and restaurants;
D. Social contact
  - Each family (or anyone living together with others) may meet up with a maximum of 5 people. These must always be the same people;
  - If you can respect the distance of 1.5 m, you can do activities with a maximum of 10 people, e.g. walking or cycling;
E. Sports and leisure
  - All locations have reopened (e.g. libraries, theme parks, playgrounds);
  - For official events, a maximum of 100 people inside / 200 people outside are allowed. Each organization has specific rules;
  - Camps for children are allowed
  - Wearing a face mask is mandatory in the following places: shops and shopping malls, shopping streets, crowded places, public buildings, markets, public transport, libraries, cinemas, museums, theatres, concert halls, conference halls, auditoria, fairgrounds and religious buildings;
  - You must leave your contact details when visiting a wellness centre, sports lessons in a club, swimming pools, casinos, party and reception rooms;
  - Discotheques and night clubs are not yet allowed to reopen
  - Big events are not allowed
  - Sports:
    - If you are part of a club, you are allowed to exercise together with a maximum of 50 people;
    - You can exercise in a fitness club; sports club or swimming pool;
    - You can visit a sauna or wellness centre. Publicly accessible jacuzzis, hammams and steam rooms remain closed;
  - Religion:
    - Worship services are allowed
    - A maximum of 100 people is allowed
    - Physical contact is not allowed
    - Wearing a face mask is mandatory
F. Nurseries and schools
  - Nurseries are open and your nursery will provide more information;
  - Your school will provide more information about the new academic year.

## Appendix 2

### Study questionnaire

A. **DEMOGRAPHIC QUESTIONS**

1. As which gender do you identify?
  □ male.
  □ female.
  □ other
2. How old are you? (numeric value)
3. In which province do you live? (drop down list)
4. Do you currently work?

- Yes
- No, I am incapacitated
- No, I am (pre-)pensioned
- No, I am unemployed
- No, I am a student
- No, I am a homemaker
- No, I have never or not yet worked

5. What is the highest level of education you have completed?

- None
- Primary education
- Lower secondary education (1st 3 years)
- Higher secondary education (minimum 6 years)
- Higher non-university education / professional bachelor
- University / academic bachelor, master or doctorate

6. Besides you, how many people currently live in your household and how old are they?yes/no if yes > add person + age in table … … … (numeric value)

- + None
- Add person (button)

1. Age: … … … (numeric value)
2. What is your household’s average annual income after taxes (in EUR)?

- Less than €15.000
- Between €15.000 and €29.999
- Between €30.000 and €44.999
- More than €45.000

8. Please indicate your proficiency in the following languages. If you are skilled in one or more additional languages, not presented in the list below, please add these as well (maximum 3).

**Table:**

	Native	Fluent	Intermediate	Basic	None
Dutch	□	□	□	□	□
French	□	□	□	□	□
German	□	□	□	□	□
English	□	□	□	□	□
Other: … … … … … (text)	□	□	□	□	□

B. **HEALTH SITUATION**

9. How would you rate your health today? (numeric slider 0–100, labeled “very bad” and “very good” at the ends)
10. How often do you depend on someone’s help or care to maintain your health and wellbeing (e.g. grocery shopping, washing, medical care)?

- I am not dependent on someone’s help or care
- Less than once a month
- 1 to 3 times a month
- 1 to 3 times a week
- More than 4 times a week

➔ skip 10 only if answer is “I am not dependent on someone’s care”

11. Why are you dependent on someone else’s help or care? (multiple answers possible)

- Because of my age
- I have difficulty walking or moving
- I have a physical condition
- I have a mental condition
- Other: (text)

12. Do you have someone close to you (e.g. family member, good friend) who is dependent on your help or care to maintain their health and wellbeing (e.g. grocery shopping, washing, medical care)?

- No
- Yes

➔ If answer is “yes”, then ask 12:

13. Why is that person dependent on your help or care? (multiple answers possible)

- Because of his/her age
- He/she has difficulty walking or moving
- He/she has a physical condition
- He/she has a mental condition
- Other: (text)

C. **RISK PERCEPTION AND VULNERABILITY TO COVID-19**

14. Since the beginning of the pandemic, have you tested positive for COVID-19? (multiple answers possible, but not in combination with “No, I did not test positive”)

- No, I did not get tested and did not have any symptoms matching COVID-19
- No, but I had symptoms that matched with COVID-19
- Yes, but I did not have symptoms
- Yes, I was ill but not hospitalised
- Yes, I was ill and hospitalised

➔ If answer is “no”, then ask 14 and skip 15; if answer is “No, but I had symptoms that matched with COVID-19” or “Yes, …” then skip 14 and ask 15

15. How would you rate the consequences for your health, should you become infected with the virus of COVID-19? (numeric slider 0–100, labeled “not at all severe” and “very severe” at the ends)
16. How did you experience the consequences for your health when you were infected with the virus of COVID-19? (numeric slider 0–100, labeled “not at all severe” and “very severe” at the ends)
17. Did someone close to you (e.g. relative, good friend, close colleague) test positive for COVID-19? (multiple answers possible)

- No
- No, but I know someone who had symptoms that matched with COVID-19
- Yes, someone who was ill and hospitalised
- Yes, someone who was ill but not hospitalised
- Yes, but he/she did not have symptoms

18. In your opinion, what is the risk of you or someone close to you (e.g. relative, good friend, close colleague) becoming infected with the virus of COVID-19?

**Table:**

	No risk	Unlikely	Neutral	Likely	Definite	Not applicable
Yourself	□	□	□	□	□	□
Your parents	□	□	□	□	□	□
Your grandparents	□	□	□	□	□	□
Your partner	□	□	□	□	□	□
Your child (ren)	□	□	□	□	□	□
A good friend	□	□	□	□	□	□
A close colleague	□	□	□	□	□	□
Other: … … (text)	□	□	□	□	□	□

D. **SOURCES OF INFORMATION, TRUST AND UNDERSTANDING**

19. To what extent do you consider yourself informed about the current COVID-19 measures? (numeric slider 0–100, labeled “not at all informed” and “very well informed” at the ends)
20. Which **channel** do you prefer to use for accessing information on COVID-19 measures? (multiple answers possible)

- None
- Television
- Radio
- Newspaper or news site
- Social media
- Other: … … (text)
- I do not know

21. To what extent have the listed groups contributed in informing you about the current COVID-19 measures?

**Table:**

	Not at all	Slightly	Moderately	Very	Entirely	Not applicable
Politicians (e.g. national security council)	□	□	□	□	□	□
Experts (e.g. expert committee GEES, doctor, epidemiologist)	□	□	□	□	□	□
Journalists	□	□	□	□	□	□
Close contacts (e.g. relative, friend)	□	□	□	□	□	□
Others, please state (e.g. influencers, etc.): … … (text)	□	□	□	□	□	□

## To what extent do you consider the information from the listed groups about the current COVID-19 measures to be clear?

**Table:**

	Completely unclear	Rather unclear	Neutral	Rather clear	Completely clear	Not applicable
Politicians (e.g. national security council)	□	□	□	□	□	□
Experts (e.g. expert committee GEES, doctor, epidemiologist)	□	□	□	□	□	□
Journalists	□	□	□	□	□	□
Close contacts (e.g. relative, friend)	□	□	□	□	□	□
Others, please state (e.g. influencers, etc.): … … (text)	□	□	□	□	□	□

## To what extent do you consider the information from the listed groups about the current COVID-19 measures trustworthy?

**Table:**

	Very untrustworthy	Rather Untrustworthy	Neutral	Rather trustworthy	Very trustworthy	Not applicable
Politicians (e.g. national security council)	□	□	□	□	□	□
Experts (e.g. expert committee GEES, doctor, epidemiologist)	□	□	□	□	□	□
Journalists	□	□	□	□	□	□
Close contacts (e.g. relative, friend)	□	□	□	□	□	□
Others, please state (e.g. influencers, etc.): … … (text)	□	□	□	□	□	□

E. **BEHAVIOUR CONCERNING CURRENT COVID-19 MEASURES**

22. To what extent do you understand the listed COVID-19 measures that apply during August?

**Table:**

	Not at all	Slightly	Moderately	Well	Very well	Not applicable
The social bubble is limited to 5 people	□	□	□	□	□	□
Private events are limited to max. 10 people	□	□	□	□	□	□
Officially organized events are limited to 200 people indoors and 400 people outdoors	□	□	□	□	□	□
Working from home is strongly recommended if possible	□	□	□	□	□	□
Shop with max. One other person	□	□	□	□	□	□
Wearing a face mask is required in public spaces where it is mandatory (e.g. shopping streets, cinemas) and when keeping 1.5 m distance is not possible	□	□	□	□	□	□
A form needs to be filled when returning or travelling to Belgium from abroad	□	□	□	□	□	□
Three types of travel zones (red, orange, green) exist that determine whether travel is allowed and if quarantine or testing is required upon return	□	□	□	□	□	□

## To what extent do you consider each of the listed measures that apply during august useful to prevent the further spread of COVID-19?

**Table:**

	Not at all	Slightly	Moderately	Rather useful	Extremely	Not applicable
The social bubble is limited to 5 people	□	□	□	□	□	□
Private events are limited to max. 10 people	□	□	□	□	□	□
Officially organized events are limited to 200 people indoors and 400 people outdoors	□	□	□	□	□	□
Working from home is strongly recommended if possible	□	□	□	□	□	□
Shop with max. One other person	□	□	□	□	□	□
Wearing a face mask is required in public spaces where it is mandatory (e.g. shopping streets, cinemas) and when keeping 1.5 m distance is not possible	□	□	□	□	□	□
A form needs to be filled when returning or travelling to Belgium from abroad	□	□	□	□	□	□
Three types of travel zones (red, orange, green) exist that determine whether travel is allowed and if quarantine or testing is required upon return	□	□	□	□	□	□

## Would you consider the following statements to be correct interpretations of the COVID-19 measures that apply during august?

**Table:**

	True	False	Don’t know
A household of 2 is allowed to organize a party or weekend trip with 10 other adults	□	□	□
When you meet a colleague after work for drinks and you maintain 1.5 m distance, this person is not part of your household’s bubble of 5	□	□	□
Your household’s bubble of 5 can include people living in another city	□	□	□
It is not mandatory to wear a face mask while exercising	□	□	□
It is mandatory to wear a face mask when you go for a walk in a park or forest	□	□	□
When you can maintain 1.5 m distance, it is not necessary to wear a face mask in public spaces	□	□	□
It is not allowed to visit a bar, indoors or outdoors, with people who are not part of your bubble of 5	□	□	□
If you visit a family that consists of 3 adults and 2 children under 12, you can still add 2 more adults to your bubble of 5	□	□	□
If you travel from an orange zone to Belgium, you do not need to be tested or quarantined upon return	□	□	□
For weddings, it is allowed to invite up to 100 guests.	□	□	□

## To what extent do you consider it easy to adhere to each of the listed COVID-19 measures that apply during august?

**Table:**

	Very difficult	Rather difficult	Neutral	Rather easy	Very easy	Not applicable
The social bubble is limited to 5 people	□	□	□	□	□	□
Private events are limited to max. 10 people	□	□	□	□	□	□
Officially organized events are limited to 200 people indoors and 400 people outdoors	□	□	□	□	□	□
Working from home is strongly recommended if possible	□	□	□	□	□	□
Shop with max. One other person	□	□	□	□	□	□
Wearing a face mask is required in public spaces where it is mandatory (e.g. shopping streets, cinemas) and when keeping 1.5 m distance is not possible	□	□	□	□	□	□
A form needs to be filled when returning or travelling to Belgium from abroad	□	□	□	□	□	□
Three types of travel zones (red, orange, green) exist that determine whether travel is allowed and if quarantine or testing is required upon return	□	□	□	□	□	□

## To what extent have you adhered to each of the listed COVID-19 measures that apply during august?

**Table:**

	Not at all	Slightly	Moderately	Quite well	Completely	Not applicable
The social bubble is limited to 5 people	□	□	□	□	□	□
Private events are limited to max. 10 people	□	□	□	□	□	□
Officially organized events are limited to 200 people indoors and 400 people outdoors	□	□	□	□	□	□
Working from home is strongly recommended if possible	□	□	□	□	□	□
Shop with max. One other person	□	□	□	□	□	□
Wearing a face mask is required in public spaces where it is mandatory (e.g. shopping streets, cinemas) and when keeping 1.5 m distance is not possible	□	□	□	□	□	□
A form needs to be filled when returning or travelling to Belgium from abroad	□	□	□	□	□	□
Three types of travel zones (red, orange, green) exist that determine whether travel is allowed and if quarantine or testing is required upon return	□	□	□	□	□	□

## To what extent do you intend to adhere in the future to each of the listed COVID-19 measures that apply during august, until new measures are issued?

**Table:**

	Not at all	Slightly	Moderately	Very	Completely	Not applicable
The social bubble is limited to 5 people	□	□	□	□	□	□
Private events are limited to max. 10 people	□	□	□	□	□	□
Officially organized events are limited to 200 people indoors and 400 people outdoors	□	□	□	□	□	□
Working from home is strongly recommended if possible	□	□	□	□	□	□
Shop with max. One other person	□	□	□	□	□	□
Wearing a face mask is required in public spaces where it is mandatory (e.g. shopping streets, cinemas) and when keeping 1.5 m distance is not possible	□	□	□	□	□	□
A form needs to be filled when returning or travelling to Belgium from abroad	□	□	□	□	□	□
Three types of travel zones (red, orange, green) exist that determine whether travel is allowed and if quarantine or testing is required upon return	□	□	□	□	□	□

## To what extent do you agree with the following statements?

**Table:**

	Strongly disagree	Disagree	Neutral	Agree	Strongly agree	Not applicable
I believe the government should oblige the public to adhere to the COVID-19 measures	□	□	□	□	□	□
I believe the government should recommend, but not oblige, the public to adhere to the COVID-19 measures	□	□	□	□	□	□
I believe it is helpful if the environment reminds me of the current COVID-19 measures (e.g. stickers on the floor)	□	□	□	□	□	□

## Appendix 3

**Table 5:** Internal consistency of questionnaire domains

	Number of items	Cronbach’s alpha	Cronbach’s alpha based on standardized items
Vulnerability	7	.897	.897
Response efficacy	8	.891	.891
Self-efficacy	8	.847	.848
Past adherence	8	.909	.912
Future adherence	8	.928	.932

## Abbreviations

COVID-19: Coronavirus disease 2019
IPCM: Infection Prevention and Control Measures
PMT: Protection Motivation Theory
